# Senescence and Senolytics: State of the Art on Cellular Senescence, Senolytics, and Healthspan

**DOI:** 10.1093/geroni/igaa057.2654

**Published:** 2020-12-16

**Authors:** Judith Campisi

## Abstract

Cellular senescence is a cell fate decision that is made by many mammalian cell types in response to damage, stress or certain physiological signals. Senescent cells arrest proliferation, essentially permanently, and develop a complex multi-component senescence-associated secretory phenotype (SASP). Recent studies using human and rodent cells, tissue samples and transgenic mouse models have defined a causal role for senescent cells, acting largely through the SASP, in a surprisingly large and diverse number of age-related diseases. Subsequently, several synthetic and natural compounds have been identified that have the ability to selectively kill senescent, but not non-senescent, cells. These compounds, termed senolytics, are now of intense interest to both basic research groups and biotechnology companies because they hold promise for postponing, ameliorating or, in some cases, reversing certain age-related pathologies. A related group of compounds, termed senomorphics, hold similar promise and act by selectively suppressing certain modules of the SASP. This symposium will feature presentations on some of the latest developments in the fields of cellular senescence and the SASP and how these cellular responses affect organismal health span. The symposium will particularly emphasize recent findings on the identification and activities of senolytic and senomorphic agents that have the potential to significantly extend the health span of mammalian organisms.

